# Endometriosis-Related Pleural Effusion: A Case Report and a PRISMA-Compliant Systematic Review

**DOI:** 10.3389/fmed.2021.631048

**Published:** 2021-03-30

**Authors:** Ping Wang, Zhilan Meng, Yakun Li, Zuojun Xu

**Affiliations:** ^1^Department of Pulmonary and Critical Care Medicine, Peking Union Medical College Hospital, Chinese Academy of Medical Science & Peking Union Medical College, Beijing, China; ^2^Department of Pathology, Peking Union Medical College Hospital, Chinese Academy of Medical Science & Peking Union Medical College, Beijing, China; ^3^Department of Pulmonary and Critical Care Medicine, Baoding No. 1 Hospital, Baoding, China

**Keywords:** thoracic endometriosis syndrome, endometriosis-related pleural effusion, clinical features, diagnosis, treatment, hemorrhagic pleural effusion

## Abstract

**Background:** Endometriosis-related pleural effusion (PE) is a relatively rare but treatable cause of bloody PE. The clinical characteristics and outcome of patients with endometriosis-related PE remain unknown.

**Objectives:** We present a case of endometriosis-related PE diagnosed on pleural fluid cytology. A systematic review of all endometriosis-related PE cases in literature was conducted to elucidate the clinical characteristics, explore the diagnostic yield of pathological examinations, and to summarize the outcomes of therapeutic approaches in this disease.

**Methods:** A case of endometriosis-related PE diagnosed in study hospital was reported. PubMed, Web of Science, and EMBASE were searched for publications pertaining to cases of endometriosis-related PE using predefined search terms. This case and those identified from PubMed, Web of Science, and EMBASE were analyzed.

**Results:** A total of 67 patients were included. Catamential symptoms were presented in 30 (44.8%) patients. Dyspnea was presented in 50 patients (74.6%), followed by right chest/shoulder pain in 34 (50.7%) and cough in 18 (26.9%). 82.8% of the patients had concomitant pelvic endometriosis and 76.7% was infertile or nulliparous. The diagnostic yield of pleural fluid cytological examination, percutaneous pleural biopsy, and surgical biopsy was 9.0, 45.5, and 78.7%, respectively. The patients who received surgery-based therapy had a significantly longer time to relapse than those who received progestational agents or GnRH analogs alone (*P* = 0.025) or hysterectomy and bilateral salpingoophorectomy (HBSO) (*P* = 0.040).

**Conclusions:** High clinical awareness of pleural endometriosis is essential in all female with hemorrhagic PE, especially in young females who have infertility and/or pelvic endometriosis. Plerual fluid cytology might be a simple minimally invasive and cost-effective modality in the diagnosis of endometriosis-related PE. Treatment is challenging due to high recurrence and the optimal management of endometriosis-related PE needs further evaluation. The combined approach by surgery and hormonal therapy may achieve the best relapse-free survival.

## Introduction

Common causes of bloody pleural effusion include trauma, iatrogenesis, and malignancy. Thoracic endometriosis syndrome (TES) is a rare disorder characterized by the presence of functioning endometrial tissue in pleural, lung parenchyma, and airway (1). A majority of patients with TES present catamenial pneumothorax (73%), while ~14% of the cases show hemothorax (2). Although endometriosis-related pleural effusion(PE) is a benign and treatable disease, it is important to take it into consideration especially in women of childbearing age. The clinical characteristics of TES have been reviewed previously (2, 3). These reviews primarily focused on catamenial pneumothorax or catamenial chest pain. The clinical features of endometriosis-related PE are limited. Diagnosis of endometriosis-related PE is challenging and depends on cytological and/or histopathological examinations demonstrating endometrial cells in pleural fluids (PF) or tissue. However, there were no data examining the diagnostic yield of diagnostic options for endometriosis-related PE. Currently, there is no standard treatment for endometriosis-related PE. Treatment options include hormonal therapy [progestational agents, danazol, and gonadotropin-releasing hormone (GnRH) analogs], thoracic surgery [removal of ectopic endometrial tissue, closing diaphragmatic defects, pleurectomy and pleurodesis through video-assisted thoracoscopic surgery (VATS) or thoracotomy], hysterectomy and bilateral salpingoophorectomy (HBSO), and combined therapies. Evaluations of the outcomes of PE treatments are lacking. Here, we report a case of endometriosis-related PE. Endometriosis in this case was confirmed by PF cytological examinations. We review the clinical symptoms, radiological findings, gynecologic and obstetric concomitants, and diagnostic accuracy of pathological examinations in published PE cases. Finally, we summarize the outcomes of therapeutic approaches in patients with endometriosis-related PE. Addressing these questions may improve the current understanding of diagnosis and management of this rare but important disease.

## Materials and Methods

This review conforms to the “Preferred Reporting Items for Systematic Reviews and Meta-Analyses” (PRISMA) statement (4).

### Search Strategy and Selection Criteria

#### Identification

A search of PubMed, Web of Science, and EMBASE was conducted using the key words “endometriosis AND pleural effusion” OR “catamenial haemothorax” OR “endometriosis AND haemothorax” OR “endometriosis AND hemopneumothorax” OR “catamenial hemopneumothorax” in the title and abstract on February 2020.The search yielded 97 abstracts from PubMed, 81 abstracts from Web of Science, and 98 abstracts from EMBASE.A total of 163 publications were obtained after the removal of duplicates in various sources.

#### Screening and Eligibility

**Figure 1:**
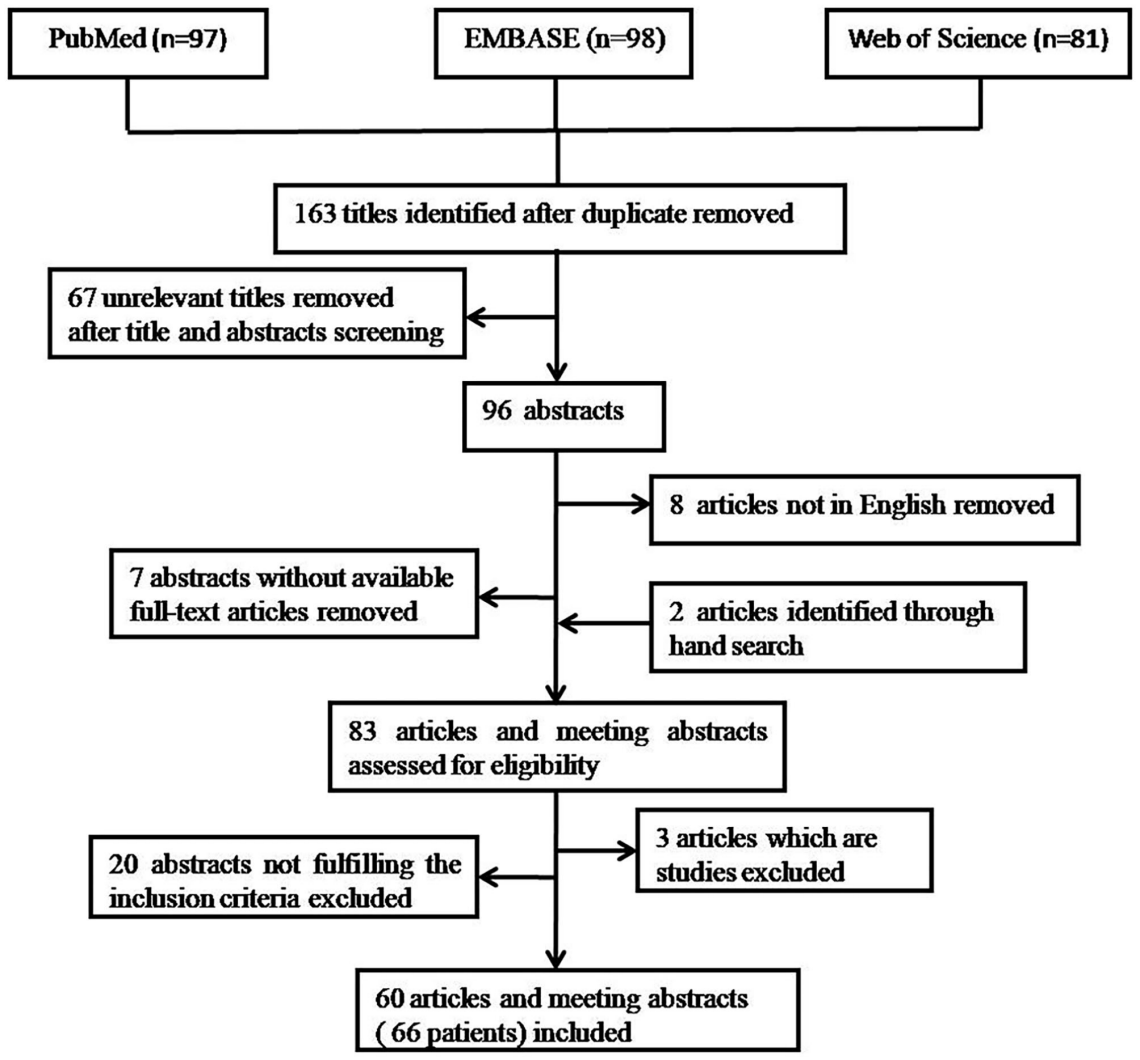
Procedure of publication retrieval and inclusion and exclusion of cases displayed in a flow chart.

Titles and abstracts were reviewed independently by two authors (PW and YL). Disagreements were resolved by discussions between the two reviewers. Sixty seven unrelevant titles were removed at the start after title screening. In the remaining 96 titles, 7 did not have a full-text article available and 8 not in Enghlish. Then, hand searches tracking the reference from the remaining 81 articles revealed 2 further articles. Consequently, 83 articles and meeting abstracts were assessed for eligibility. Only case reports or case series of endometriosis-related PE were included. The cases required to be diagnosed as endometriosis-related PE based on (1) either cytological and/or histopathological evidence of endometrial cells in PF or pleural tissue (pathological diagnosis), or (2) the finding of catamenial symptomatology, the presence of hemorrhagic PE, the presence of pelvic endometriosis, the exclusion of other causes of hemorrhagic PE, and the response to hormonal and/or surgical treatment (clinical diagnosis). Three articles which are studies were excluded. Twenty abstracts that did not fulfill the inclusion criteria were excluded. Finally, 60 articles and meeting abstracts with a total of 66 cases were included. The procedure of publication retrieval and the inclusion and exclusion of cases was illustrated in a flow chart (Figure 1).

#### Data Extraction

The following data was extracted from eligible cases and recorded on a standard data extraction form: Age, symptoms, duration between onset of symptoms and final diagnosis, past medical history, marital and reproductive history, chest CT findings, PF and VATS findings, treatment and relapse event. Treatments were classified as progestational agents or GnRH analogs, danazol, surgery-based therapy (thoracic surgery combined with or without hormonal therapy), and HBSO.

#### Quality Assessment

The methodological quality of case reports was assessed using the Joanna Briggs Institute (JBI) critical appraisal checklist for case reports independently by two authors (PW and YL). Based on this tool, the 66 cases had a low risk of bias and were included.

### Statistical Analysis

All data analyses were conducted using Statistical Package for the Social Sciences, version 17.0 for Windows (SPSS Inc., Chicago, IL, USA). Kaplan–Meier survival curves were constructed, and the significance of survival stratification was tested using the log-rank and Breslow test. Time to recurrence was calculated between the date of the treatment and the date of the recurrence of symptoms and haemothorax. *P*-values <0.05 were considered statistically significant.

## Case Presentation

**Figure 2:**
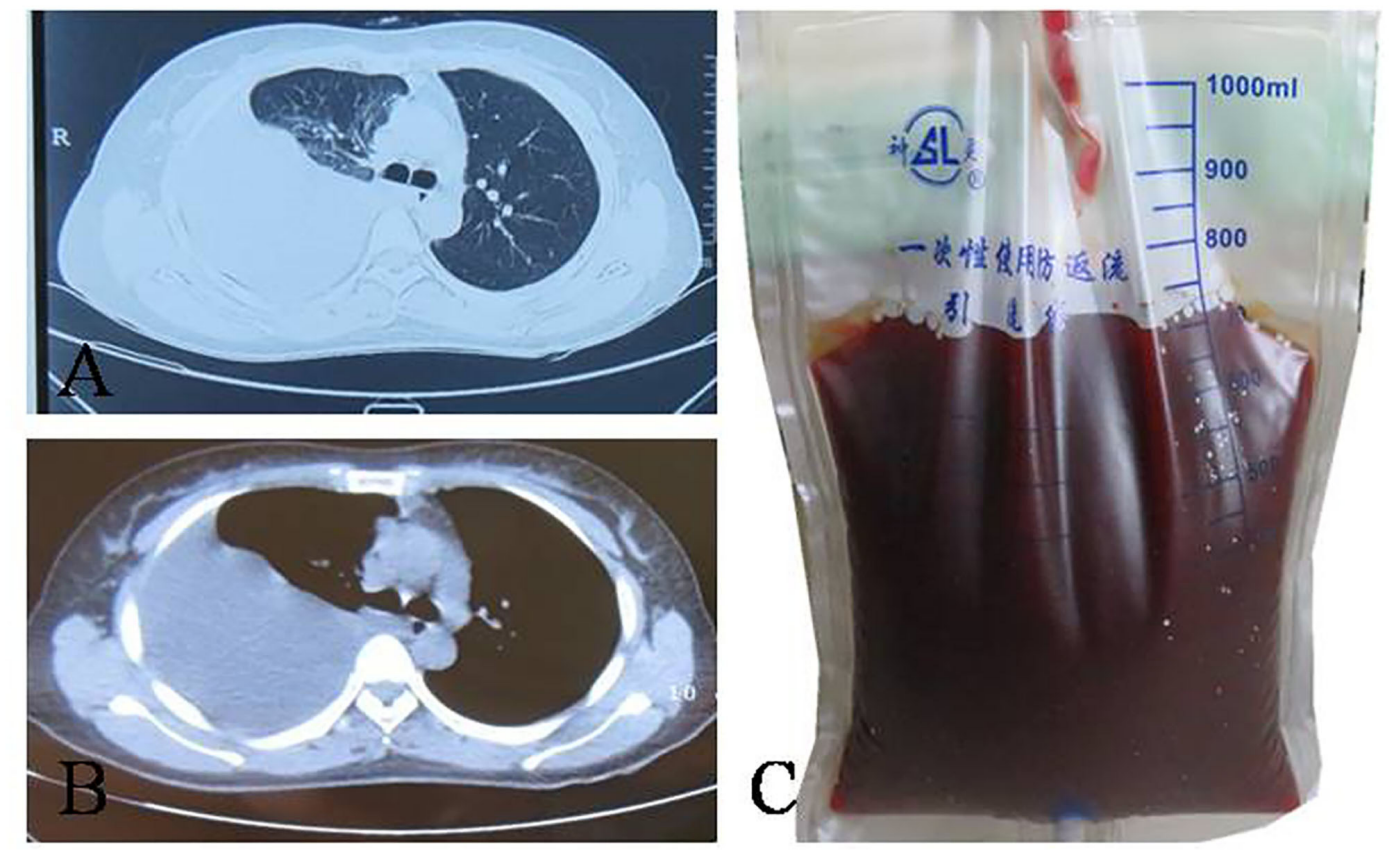
Computed tomography manifestation and pleural fluid of the patient. **(A,B)** right-sided pleural effusion; **(C)** haemorragic fluid aspirated from right pleural cavity.

**Figure 3:**
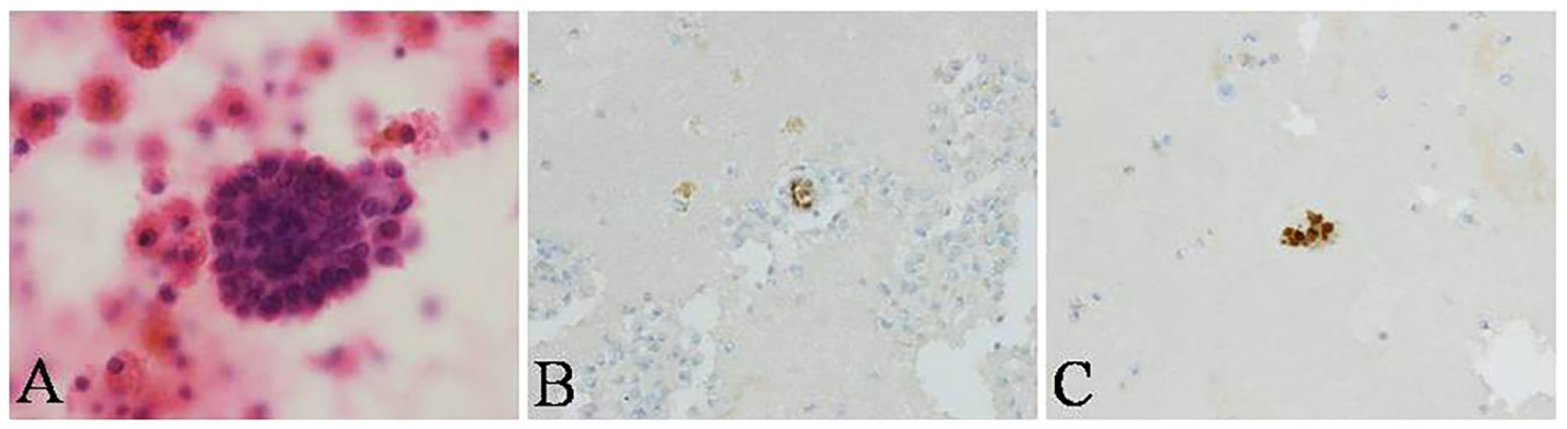
Cytological examination of pleural fluid in the patient. **(A)** scattered clusters of endometrial glandular cells (ThinPrep^TM^, H&E stain, ×400) in pleural fluid, showing nuclear positivity for **(B)** estrogen receptor and **(C)** progesterone receptor with immunohistochemical staining (conventional smear, ×400).

A 31-year-old woman was admitted to our hospital with complaints of 4 months of breathlessness. She has noticed the onset of breathlessness 4 months ago and was admitted to the local hospital. Routine blood examination revealed a moderate degree of anemia (Hemoglobin 75g/L). A massive right-sided PE was identified on chest computerized tomography (CT) scans. She underwent thoracentesis. 3,000 ml hemorrhagic fluids were drained and contained no malignant cells. Breathlessness was relieved. She was discharged with a diagnosis of hemorrhagic pleural effusion of unknown origin. The patient was transferred to our hospital because of recurrence of dyspnea 2 months ago. She was a non-smoker and had a 4-year history of infertility and bilateral ovarian chocolate cysts. Two times of laparoscopic ovarian cystectomy have been done in the past 4 years. She had regular periods every 28 days, lasting 7 days with severe dysmenorrhoea. The physical examination was consistent with massive right-sided PE. A repeated Chest CT scan revealed massive right PE (Figures 2A,B). Thoracentesis and chest tube drainage were performed. Pleural aspirates revealed a deep red-colored hemorrhagic appearance (Figure 2C). Blood laboratory findings revealed anemia: hemoglobin 75g/l, red blood cells 3.9 × 10^12^/L, hematocrit 31%. Analysis of the pleural fluids showed the following values: red blood cell 260,000/mm^3^, hematocrit 2%, white blood cell 190/mm^3^, mononuclear cells 84%, total protein 50 g/L, albumin 33g/l, lactate dehydrogenase 260 IU/l, adenosine deaminase 15.5 IU/l, glucose 5.2 mmol/L, T-SPOT.TB 0 spot forming cells/10^6^ PBMCs, normal carcinoembryonic antigen, negative tuberculosis/non-tuberculous mycobacterium DNA amplification, negative acid-fast bacilli staining, and negative culture for bacteria, fungi, and mycobacteria. Cytological examination of liquid based cytology (ThinPrep^TM^) smears showed scattered clusters of endometrial glandular cells (Figure 3A). Immunohistochemical staining of sediment cell blocks for estrogen receptor and progesterone receptor showed nuclear positivity (Figures 3B,C). A diagnosis of endometriosis-related PE was made. The patient was prescribed leuprolide (3.6 mg intramuscularly every month). At 14-month follow-up, there was no recurrence of breathlessness. Chest radiography showed a little right-sided pleural effusion. The patient underwent HBSO because of severe dysmenorrhoea, menorrhagia, and infertility. Oral Tibolone Tablets were prescribed (1.25 mg every day). Spontaneuos right-sided hydropneumothorax occurred 3 months later. The patient discontinued Tibolone tablets and the symptoms of cough and dyspnea improved. She declined repeated chest CT, thoracentesis, and thoracic surgery.

## Results

Including this case, we analyzed a total of 67 patients with endometriosis-related PE (5–64). Demographic information and clinical characteristics were shown in Table 1. The mean age at presentation was 35 years old. The median duration between onset of symptoms and final diagnosis was 6 months. Catamenial symptoms were presented in 30 (44.8%) patients. Dyspnea was presented in 50 (74.6%) patients followed by right chest/shoulder pain in 34 (50.7%) and cough in 18 (26.9%). Detailed descriptions of chest CT manifestations in endometriosis-related PE were provided in 58 cases. Pneumothorax was found in 24 (35.8%), lung bullae in 1 (1.7%), and pleural thickening or nodularity in 5 (8.6%).

**Table 1:** Demographics and clinical characteristics of patients with endometriosis-related pleural effusion (*n* = 67).

	**Data**
Age (years)^§^	35 ± 8
Respiratory symptomatic	60 (89.6%)
Catamential	30 (44.8%)
Dyspnea	50 (74.6%)
Right chest/shoulder pain	34 (50.7%)
Cough	18 (26.9%)
Duration (months)^*^	6 (10)
Chest CT findings	
Hemithorax involvement of pleural effusion	
Unilateral right-sided	53 (79.1%)
Bilateral	14 (20.9%)
Pneumothorax^†^	24 (35.8%)
Lung bullae ^‡^	1 (1.7%)
Pleural thickening/nodularity^‡^	5 (8.6%)

*Data are presented as mean± standard deviation or median (interquartile range) or number (%)*.

§ Age was available in 65 patients;

* Duration was available in 53 patients;

† pneumothorax was available in 64 patients;

‡ *lung bullae and pleural thickening/nodularity was available in 58 patients*.

Of the 43 patients with available data of obstetric concomitants, 33 (76.7%) were infertile or nulliparous. History of gynecologic concomitants was reported in 57 patients. Twenty nine (50.9%) had dysmenorrhoea and 12 (21.4%) received pelvic surgery. Pelvic endometriosis was found in 48 (82.8%) of 58 patients with available data of pelvic examination.

Pathological diagnosis was achieved in 44(65.7%) patients and clinical diagnosis in 23 (34.3%) patients. A cytological examination of PF was performed in all patients. Percutaneous pleural biopsy was performed in 11 patients and surgical biopsy in 47 patients. The diagnostic yields were 6 (9.0%) patients, 5 (45.5%) patients, and 37 (78.7%) patients, respectively.

Of the 41 patients with available information of treatments and follow-ups, 27 received surgery-based therapy, 13 received progestational agents or GnRH analogs, 4 received danazol, and 7 received HBSO (8 patients received more than one therapy sequentially in the follow-up period). Of the 27 patients who received thoracic surgery interventions, pleurodesis was performed in 11 (40.7%) cases, pleurectomy in 10 (37.0%), decortication in 8 (29.6%), repair of diaphragmatic fenestrations in 8 (29.6%), lung wedge resection in 4 (14.8%), and coverage of lung surface with polyglycolic acid sheets in 1 (3.7%) (12 patients received more than one procedure during a thoracic surgery intervention). The mean follow-up period of each treatment was 16 months (ranging 2–126 months). There were 13 relapse events. The median time of relapse was 55 months in patients with surgery-based therapy, 17 months in patients with progestational agents or GnRH analogs, and 9 months in patients with HBSO. Patients who received surgery-based therapy had a significantly longer time of relapse than those who received progestational agents or GnRH analogs alone (*P* = 0.025) or HBSO (*P* = 0.040).

## Discussion

Hemothorax related to endometrial tissue in pleura known as endometriosis-related PE is an important cause of bloody PE in women. To the best of our knowledge, this is the first review that summarizes clinical characteristics, diagnosis, and management of patients with endometriosis-related PE.

In this review, the average age at presentation was 35 years old, similar to 34–37 years old in TES cohorts in which a majority of pathologic entity is catamenial pneumothorax (2, 65, 66). Dyspnea was the most common symptom while chest pain and cough were relatively uncommon. Additionally, no haemoptysis was seen in patients with endometriosis-related PE. In contrast, previous review by Channaabasavaiah et al. reported that chest pain is the most common symptoms (90%) while dyspnea (31%), haemoptysis (7%), and cough (rare) are less common (2). This discrepancy can be explained by different pathological entities in the two study cohorts. Although the symptoms of TES often developed within 24–72 h of the onset of menstruation, the temporal association between symptoms and menses was not apparent in patients with endometriosis-related PE. Less than half patients presented catamenial symptoms. This may be caused by insidiousness and persistence of symptoms due to accumulation of bloody pleural effusion in thoracic cavity after menstruation.

About 80% of the patients with endometriosis-related PE had concomitant pelvic endometriosis and were infertile or nulliparous. Less than one fourth patients had history of pelvic surgery. In contrast, it has been reported that patients with lung parenchymal endometriosis tended not to have pelvic disease (only a 10% association) (67) but to have a significantly higher incidence of previous vaginal delivery or gynecological operative procedures than patients with pleural endometriosis (68, 69). These findings imply that specific mechanisms maybe involved in the development of various types of TES. In the endometriosis-related PE, transdiaphagmatic pass of endometrium tissue and local metaplasia of coelomic epithelium have been thought as possible reasons (68, 70). In parenchymal type thoracic endometriosis, haematogenous expansion of endometrium tissue and microembolization after surgical operations may hold the responsibility (71–73).

Diagnosis of endometriosis-related PE is challenging and often be delayed. The median duration of symptoms before diagnosis in our cohort was 6 months. The reasons of delayed diagnosis included failure to recognize this rare disease, the non-specificity of symptoms and radiological abnormalities, and the infrequency of pathological evidence. As shown in our review, concomitant pleural or lung infiltrate in Chest CT was rarely seen in patients with endometriosis-related PE. About one third patients in our cohort did not have pathological confirmation of endometriosis diagnosis. Therefore, high clinical awareness of pleural endometriosis would be essential for all female with hemothorax, particularly in young females who have infertility and/or pelvic endometriosis. Although only 9.0% of the patients with endometriosis-related PE taken to cytological examinations resulted in a pathological diagnosis, endometrial cell clusters were found in PF samples in our case. A possible explanation would be that we performed thoracentesis and chest tube drainage at the beginning of menstruation, collected PF each day during menstruation, and informed the cytopathologist a suspicion of endometriosis-related hemothorax. In literature, a PF cytologic examination was considered to be rarely helpful (9). However, our finding confirmed the opinion that endometriosis can be reliably and safely diagnosed in cytologic materials (45, 74), highlighting the role of PF cytology in the diagnosis of endometriosis-related PE. PF cytology might be a simple, minimally invasive, and cost-effective modality in the diagnosis of endometriosis-related PE by experienced cytopathologists if it is performed in appropriate time.

Joseph and Sahn reported a 62% recurrence rate in hormonal therapy compared to 25% in surgical pleurodesis at 1 year in patients with catamenial hemothorax, and 60% cases initially treated with hormone therapy required surgical treatment for recurrence (75). However, whether or not treatment options were related to recurrence-free survival in endometriosis-related PE was not examined. We found that surgery-based therapy has a longer time to recurrence compared with hormonal therapy or HBSO in patients with endometriosis-related PE. TES is a spectrum of diseases with hemothorax representing the late manifestation, which implicates the presence of a high burden of proliferating parietal and visceral pleural implants. In addition, diaphragmatic fenestrations are more prevalent in catamenial hemothorax than catamenial pneumothorax cases (71 vs. 26%) (75). It is plausible to predict that surgical treatment including removal of ectopic endometrial tissue, closing diaphragmatic defects, abrading of pleural surfaces, pleurodesis and pleurectomy would be more effective in preventing ongoing recurrences. As HBSO requires estrogen replacement in women of reproductive age, early adding back estrogen therapy after HBSO may cause the reactivation of thoracic endometrial tissue and relapse of the disease, as demonstrated in our case.

There are several limitations to this systematic review. First, we excluded articles that were either in a language other than English or without full text available. Therefore, we might have missed some key cases of high relevance. Second, our study design was a retrospective review of the cases reported in literature, and a selection bias should therefore be acknowledged. Moreover, not all reports gave sufficient detail regarding the radiological findings and treatment follow-up data. Third, the sample size of our study was small because endometriosis-related PE is a rare disease. Further prospective studies with larger sample sizes are therefore needed.

## Conclusions

Physicians should be familiar with the clinical features of this potentially treatable cause of spontaneous hemothorax. The cytologic diagnosis of endometriosis-related PE can be made if the examination is done in a right clinical setting with good clinical-pathological communications, avoiding unnecessary diagnostic surgical procedures for both patients and surgeons. Treatment is challenging due to high recurrence and the optimal management of endometriosis-related PE needs further evaluation. It appears that a combined approach of surgery and hormonal therapy may improve the relapse-free survival.

## Data Availability Statement

The original contributions presented in the study are included in the article/supplementary material, further inquiries can be directed to the corresponding author/s.

## Ethics Statement

Ethics approval and consent to participate was waived for this systematic review by the Ethics Committee of Peking Union Medical College Hospital.

## Consent for Publication

Written informed consent was obtained from the patients for publication of this case report.

## Author Contributions

PW contributed to study design, literature search, data collection, data analysis, and writing, review and approval of the final manuscript. ZM contributed to data collection, data analysis, writing, review and approval of the final manuscript. YL contributed to literature review, data extraction, review and approval of the final manuscript. ZX contributed to study design and review and approval of the final manuscript. All authors contributed to the article and approved the submitted version.

## Conflict of Interest

The authors declare that the research was conducted in the absence of any commercial or financial relationships that could be construed as a potential conflict of interest.

## Abbreviations

TES: thoracic endometriosis syndrome
PE: pleural effusion
PF: pleural fluid
GnRH: gonadotropin-releasing hormone
VATS: video-assisted thoracoscopic surgery
HBSO: hysterectomy and bilateral salpingoophorectomy
CT: computerized tomography.

## References

[1] Nezhat C Buescher E Paka C Hajhosseini B Hilaris GE. Thoracic endometriosis syndrome. World Clin Obstet Gynecol. (2011) 1:228–38.

[2] Channabasavaiah AD Joseph JV. Thoracic endometriosis: revisiting the association between clinical presentation and thoracic pathology based on thoracoscopic findings in 110 patients. Medicine. (2010) 89:183–8. 10.1097/MD.0b013e3181df67d5 20453605

[3] Bobbio A Canny E Mansuet Lupo A Lococo F Legras A Magdeleinat P. Thoracic endometriosis syndrome other than pneumothorax: clinical and pathological findings. Ann Thorac Surg. (2017) 104:1865–71. 10.1016/j.athoracsur.2017.06.049 29054304

[4] Liberati A Altman DG Tetzlaff J Mulrow C Gøtzsche PC Ioannidis JPA. The PRISMA statement for reporting systematic reviews and meta-analyses of studies that evaluate health care interventions: explanation and elaboration. J Clin Epidemiol. (2009) 62:e1–34. 10.1016/j.jclinepi.2009.06.006 19631507

[5] Pankratjevaite L Samiatina-Morkuniene D. A case report of thoracic endometriosis—a rare cause of haemothorax. Int J Surg Case Rep. (2017) 33:139–42. 10.1016/j.ijscr.2017.02.052 28315819 PMC5358902

[6] Nair SS Nayar J. Thoracic endometriosis syndrome: a veritable pandora's box. J Clin Diagn Res. (2016) 10:QR04-8. 10.7860/JCDR/2016/17668.7700 27190904 PMC4866202

[7] Mehta AA Gupta A Venkitakrishnan R. A case of young woman with recurrent right pleural effusion. Lung India. (2015) 32:648–650. 10.4103/0970-2113.168097 26664182 PMC4663879

[8] Bhattacharjee S Deb J Saha R Chakrabarti S Mukherji J Tapadar SR. Pleural endometriosis: an exceptional cause of hemorrhagic pleural effusion. J Obstet Gynaecol India. (2014) 64:100–4. 10.1007/s13224-012-0313-y 25404828 PMC4228007

[9] Sevinç S Unsal S Oztürk T Uysal A Samancilar O Kaya SO. Thoracic endometriosis syndrome with bloody pleural effusion in a 28 year old woman. J Pak Med Assoc. (2013) 63:114–6. 23865146

[10] Marchiori E Zanetti G Rodrigues RS Souza LS Souza Junior AS Francisco FA. Pleural endometriosis: findings on magnetic resonance imaging. J Bras Pneumol. (2012) 38:797–802. 10.1590/s1806-37132012000600017 23288127

[11] Peterzan M Reynolds T Dulay K Wooldridge R. Thoracic endometriosis syndrome manifesting as atraumatic haemothorax causing difficult ventilation under general anaesthesia. BMJ Case Rep. (2012) pii: bcr2012007206. 10.1136/bcr-2012-007206 23257939 PMC4543866

[12] Halvorson SA Ricker MA Barker AF Patton PE Harrison RA Hunter AJ. Thoracic endometriosis unmasked by ovarian hyperstimulation for in vitro fertilization. J Gen Intern Med. (2012) 27:603–7. 10.1007/s11606-011-1959-3 22234445 PMC3326110

[13] Nwiloh J. Diaphragmatic patch: a useful adjunct in surgical treatment of recurrent catamenial hemothorax. Rev Port Pneumol. (2011) 17:278–80. 10.1016/j.rppneu.2011.06.006 21795017

[14] Goumenou A Matalliotakis I Mahutte N Koumantakis E. Endometriosis mimicking advanced ovarian cancer. Fertil Steril. (2006) 86:219.e23–5. 10.1016/j.fertnstert.2005.12.044 16818037

[15] Lee HT Wang HC Huang IaT Chang HC Lu JY. Endometriosis associated with hemothorax. J Chin Med Assoc. (2006) 69:42–6. 10.1016/s1726-4901(09)70110-8 16447926

[16] Black H Sigal D Barnes D Felisky C Follette D Harper R. A 25-year-old patient with spontaneous hemothorax. Chest. (2005) 128:3080–3. 10.1378/chest.128.4.3080 16236990

[17] Byanyima RK. Menstruation in an unusual place: a case of thoracic endometriosis in Kampala, Uganda. Afr Health Sci. (2001) 1:97–8. 12789123 PMC2141555

[18] Moffatt SD Mitchell JD. Massive pleural endometriosis. Eur J Cardiothorac Surg. (2002) 22:321–3. 10.1016/s1010-7940(02)00277-4 12142212

[19] Rychlik DF Bieber EJ. Thoracic endometriosis syndrome resembling pulmonary embolism. J Am Assoc Gynecol Laparosc. (2001) 8:445–8. 10.1016/s1074-3804(05)60348-9 11509791

[20] Bhojawala J Heller DS Cracchiolo B Sama J. Endometriosis presenting as bloody pleural effusion and ascites-report of a case and review of the literature. Arch Gynecol Obstet. (2000) 264:39–41. 10.1007/pl00007484 10985620

[21] Flanagan KL Barnes NC. Pleural fluid accumulation due to intra-abdominal endometriosis: a case report and review of the literature. Thorax. (1996) 51:1062–3. 10.1136/thx.51.10.1062 8977611 PMC472672

[22] Myers TJ Arena B Granai CO. Pelvic endometriosis mimicking advanced ovarian cancer: presentation with pleural effusion, ascites, and elevated serum CA 125 level. Am J Obstet Gynecol. (1995) 173:966–7. 10.1016/0002-9378(95)90381-x 7573283

[23] Schlueter FJ McClennan BL. Massive ascites and pleural effusions associated with endometriosis. Abdom Imaging. (1994) 19:475–6. 10.1007/BF00206945 7950833

[24] Heneghan MA Teixidor HS. Pleuroperitoneal endometriosis. AJR Am J Roentgenol. (1979) 133:727–30. 10.2214/ajr.133.4.727 114022

[25] Charles D. Endometriosis and hemorrhagic pleural effusion. Obstet Gynecol. (1957) 10:309–12. 13477585

[26] Van der Merwe E Schuurmans MM de Kock F Siebert I Wright C Bolliger CT. Bloodstained pleural effusion in a 38-year-old non-smoking female. Respiration. (2005) 72:101–4. 10.1159/000083410 15753644

[27] Tiwari M. Gami S. Pleural endometriosis in an infertile woman: a case report. Chest. (2017) 152:4 Supplement 1 (A931). 10.1016/j.chest.2017.08.96628693778

[28] Rachid M. Mekhaiel E. Recurrent unexplained pleural effusion in young female? Think outside the Box [Abstract]. Am J Respir Crit Care Med. (2017) 195:A3496. 10.1164/ajrccmconference.2017.B44

[29] Moody A.D. Pagan R. Chen A. Nassar A. A vicious cycle: an uncommon presentation of thoracic endometriosis [Abstract]. Am J Respir Crit Care Med. (2017) 195:A5508. 10.1164/ajrccmconference.2017.C42

[30] Merriman S.R. Gupta N. Thoracic endometriosis presenting as catamenial hemopneumothorax [Abstract]. Am J Respir Crit Care Med. (2017) 195.

[31] Williams JA Shannon V. A different kind of premenstrual syndrome [Abstract]. Am J Respir Crit Care Med. (2017) 195. 10.1164/ajrccmconference.2017.C46

[32] Tijskens M. Nowé V. A catamenial cause of thoracic pain [Abstract]. Acta Clin Belg. (2017) 72 Supplement 2:11–2. 10.1080/17843286.2017.1401314

[33] Nguyen J. Kamangar N. Massive hemothorax as a complication of thoracic endometriosis syndrome [abstract]. Chest. (2016) 150:1255A. 10.1016/j.chest.2016.08.1368

[34] Hashmi H. Adrish M. Chest pain in a young female-time to think differently [abstract]. Am J Respir Crit Care Med. (2015) 191:A1407. 10.1016/s0025-6196(12)64859-9

[35] Aloma A.I. Cheng G.Z. Folch E. Majid A. Catamenial hemothorax: case report and a systematic review [abstract]. Am J Respir Crit Care Med. (2015) 191:A5856.

[36] Yao S. Berkowitz E. Najjar H. Tsaltas J. Pleural endometriosis: An unexpected finding during thoracic surgery. BJOG. (2015) 122(Suppl 2):128–9. 10.1111/14710528.1337525545903

[37] Nalle M Sagubadi S Penco AJF. Catamenial hemothorax: a unique case of spontaneous diaphragmatic rupture resulting in intrathoracic colonic strangulation and necrosis[abstract]. Am J Respir Crit Care Med. (2014) 189:A6431. 10.1002/jsfa.2740310210

[38] Packard L.K Adamson G.D. Endometriosis presenting with massive ascites and pleural effusion: a case Report. J Endometr Pelvic Pain Disord. (2013) 5:123–5. 10.5301/je.5000162

[39] Chooljian D Ng J Murakami T Miyai T Chang C-H Chung J. Endometriosis as a cause of massive ascites and pleural effusion [abstract]. Chest. (2012) 142:501A. 10.1378/chest.1389454

[40] Stevenson EK Sloan KA Narsule CK Kretschman DM Sarita-Reyes CD Steiling K. Catamenial hemothorax in a patient with multiple sclerosis. Am J Respir Crit Care Med. (2014) 190:e69–70. 10.1164/rccm.201407-1283IM 25496111

[41] Carrillo J.F Singla A. Catamenial hemothorax (CH) without respiratory symptoms. A case report. J Minim Invasive Gynecol. (2012) 19:S132–S133. 10.1016/j.jmig.2012.08.351

[42] Brown C. Lee K. Diagnostic dilemma for the infectious diseases physician: the West African patient with a bloody pleural effusion [abstract]. Am J Respir Crit Care Med. (2010) 181:A6880.

[43] Mwenechanya S. Beck I. Unusual presentation of endometriosis with massive ascites and recurrent pleural effusion: A case report and review of the literature. Gynecol Surg. (2007) 4:57–9. 10.1007/s10397-006-0220-2

[44] Rawala MS Khaliq MF Iqbal MA Naqvi STS Farhan K Myers A. A rare case of cyclical hemothorax: thoracic endometriosis syndrome. Case Rep Pulmonol. (2018) 2018:9830797. 10.1155/2018/9830797 30210894 PMC6120274

[45] Gupta P Gupta N Bal A Sehgal IS Muthu V Rajwanshi A. The eyes don't see what the mind doesn't know: pleural endometriosis on effusion cytology. Cytopathology. (2018) 29:574–7. 10.1111/cyt.12601 29904975

[46] AlAqeel S AlJehani Y AlMuhaish M. Bilateral catamenial hemopneumothorax: diagnostic & management challenges. I*nt J Surg Case Rep*. (2019) 61:271–4. 10.1016/j.ijscr.2019.07.067 PMC6699417 31398668

[47] Lua LL Tran K Desai J. Refractory thoracic endometriosis syndrome with bilateral hemothorax. J Obstet Gynaecol Res. (2017) 43:1227–31. 10.1111/jog.13331 28503772

[48] Davis BM Goldstraw E Bhowmik A José RJ. A gynaecological cause of spontaneous haemopneumothorax. Br J Hosp Med. (2016) 77:602–3. 10.12968/hmed.2016.77.10.602 27723388

[49] Takahashi M Matsukura T Hirai T Mino N. Recurrent catamenial hemopneumothorax treated by coverage with polyglycolic acid sheets. J Thorac Cardiovasc Surg. (2013) 145:300–2. 10.1016/j.jtcvs.2012.08.054 22982032

[50] Nezhat C Nicoll LM Bhagan L Huang JQ Bosev D Hajhosseini B. Endometriosis of the diaphragm: four cases treated with a combination of laparoscopy and thoracoscopy. J Minim Invasive Gynecol. (2009) 16:573–80. 10.1016/j.jmig.2009.06.012 19835800

[51] Morcos M Alifano M Gompel A Regnard JF. Life-threatening endometriosis-related hemopneumothorax. Ann Thorac Surg. (2006) 82:726–9. 10.1016/j.athoracsur.2005.10.002 16863799

[52] Tsunezuka Y Oda M Moriyama H Ohshima M Kurumaya H. Thoracoscopic findings and surgical management of catamenial hemopneumothorax. Ann Thorac Cardiovasc Surg. (2006) 12:197–9. 16823334

[53] Ismail Y Kamaruzzaman A. Thoracic endometriosis: a report of two cases. Med J Malaysia. (2004) 59:279–80. 15559180

[54] Bhatia DS McFadden PM Kline RC. Recurrent catamenial hemopneumothorax. South Med J. (1998) 91:398–401. 9563438

[55] Ravindran P Raj RJ Parameswaran K. Concurrent catamenial hemothorax and hemopneumothorax. Chest. (1993) 103:646–8. 10.1378/chest.103.2.646 8432182

[56] Khan A. Recurrent non-catamenial hemopneumothorax secondary to diaphragmatic and pleural endometriosis [abstract]. Am J Respir Crit Care Med. (2019) 199:9.30130137

[57] Da Silva Almeida J Da Silva Dantas Lourenço J Dos Santos Diniz J Alecrin I Colucci F. Thoracic endometriosis: a case report [abstract]. Int J Gynecol Obstet. (2018) 143(Suppl3):766.

[58] Bell J Casablanca NM Mecca J. Oh, the places endometrium can go. J Gen Intern Med. (2018) 33(Suppl 1):580–1.

[59] Yang X Steiger D. Miyakawa L. Filopei J. A rare case of thoracic endometriosis presenting with hemopneumothorax [abstract]. Am J Respir Crit Care Med. (2018) 197:MeetingAbstracts.

[60] Harischandra T Pushpakumara HA Suvarna SH. Catamenial haemopneumothorax: a case report. Heart Surg Forum. (2010) 13(Suppl 2):S157.

[61] Picozzi G Beccani D Innocenti F Grazzini M Mascalchi M. MRI features of pleural endometriosis after catamenial haemothorax. Thorax. (2007) 62:744. 10.1136/thx.2006.071415 17687105 PMC2117275

[62] Francis M Badero OO Borowsky M Lee YC Abulafia O. Pericardial effusion, right-sided pleural effusion and ascites associated with stage IV endometriosis. A case report. J Reprod Med. (2003) 48:463–5. 12856520

[63] Dhanaworavibul K Hanprasertpong J Cheewadhanaraks S Buhachat R. Bilateral pleural endometriosis. J Obstet Gynaecol Res. (2006) 32:86–9. 10.1111/j.1447-0756.2006.00356.x 16445531

[64] Charokopos N Tsiamita M Karkoulias K Panagiota R Dougenis D Spiropoulos K. Recurrent catamenial hemothorax. Monaldi Arch Chest Dis. (2004) 61:177–9. 10.4081/monaldi.2004.699 15679013

[65] Duyos I López-Carrasco A Hernández A Zapardiel I Santiago J. Management of thoracic endometriosis: single institution experienc*e*. Eur J Obstet Gynecol Reprod Biol. (2014) 178:56–9. 10.1016/j.ejogrb.2014.03.026 24809986

[66] Visouli AN. Catamenial Pneumothorax: a rare entity? Report of 5cases and review of literature. J Thorac Dis. (2012) 4 (Suppl 1):17–31. 10.3978/j.issn.2072-1439.2012.s006 23304438 PMC3537379

[67] Foster DC Stern JL Buscema J Rock JA Woodruff JD. Pleural and endometrial endometriosis. Obstet Gynecol. (1981) 58:552–6. 7301229

[68] Honoré GM. Extrapelvic endometriosis. Clin Obstet Gynecol. (1999) 42:699–711. 10.1097/00003081-199909000-00021 10451779

[69] Jubanyik KJ Comite F. Extrapelvic endometriosis. Obstet Gynecol Clin North Am. (1997) 24:411–40. 10.1016/s0889-8545(05)70311-9 9163774

[70] Suginami H. A reappraisal of the coelomic metaplasia theory by reviewing endometriosis occurring in unusual sites and instances. Am J Obstet Gynecol. (1991) 165:214–8. 10.1016/0002-9378(91)90254-o 1853899

[71] Fonseca P. Catamenial pneumothorax: a multifactorial etiology. J Thorac Cardiovasc Surg. (1998) 116:872–3. 10.1016/S0022-5223(98)00434-6 9806395

[72] Terada Y Chen F Shoji T Itoh H Wada H Hitomi S. A case of endobronchial endometriosis treated by subsegmentectomy. Chest. (1999) 115:1475–8. 10.1378/chest.115.5.1475 10334179

[73] Park WW. The occurrence of decidual tissue within the lung: report of a case. J Pathol Bacteriol. (1954) 67:563–70. 10.1002/path.1700670229 13192581

[74] Barkan GA Naylor B Gattuso P Küllü S Galan K Wojcik EM. Morphologic features of endometriosis in various types of cytologic specimens. Diagn Cytopathol. (2013) 41:936–42. 10.1002/dc.22979 23529978

[75] Joseph J Sahn SA. Thoracic endometriosis syndrome: new observations from an analysis of 110 Cases. Am J Med. (1996) 100:164–70. 10.1016/s0002-9343(97)89454-5 8629650

